# Epidemiology, Pathophysiology, and Genetics of Primary Hyperparathyroidism

**DOI:** 10.1002/jbmr.4665

**Published:** 2022-10-17

**Authors:** Salvatore Minisola, Andrew Arnold, Zhanna Belaya, Maria Luisa Brandi, Bart L. Clarke, Fadil M. Hannan, Lorenz C. Hofbauer, Karl L. Insogna, André Lacroix, Uri Liberman, Andrea Palermo, Jessica Pepe, René Rizzoli, Robert Wermers, Rajesh V. Thakker

**Affiliations:** ^1^ Department of Clinical, Internal, Anaesthesiologic and Cardiovascular Sciences, ‘Sapienza’ Rome University Rome Italy; ^2^ Center for Molecular Oncology and Division of Endocrinology & Metabolism University of Connecticut School of Medicine Farmington CT USA; ^3^ Department of Neuroendocrinology and Bone Disease The National Medical Research Centre for Endocrinology Moscow Russia; ^4^ F.I.R.M.O. Italian Foundation for the Research on Bone Diseases Florence Italy; ^5^ Mayo Clinic Division of Endocrinology, Diabetes, Metabolism, and Nutrition Mayo Clinic Rochester MN USA; ^6^ Academic Endocrine Unit, Radcliffe Department of Medicine University of Oxford, Oxford Centre for Diabetes, Endocrinology and Metabolism (OCDEM), Churchill Hospital Oxford UK; ^7^ Nuffield Department of Women's & Reproductive Health University of Oxford Oxford UK; ^8^ Division of Endocrinology, Diabetes, and Bone Diseases & Center for Healthy Aging Technische Universität Dresden Dresden Germany; ^9^ Yale Bone Center Yale School of Medicine Yale University New Haven CT USA; ^10^ Division of Endocrinology, Department of Medicine and Research Center Centre Hospitalier de l'Université de Montréal (CHUM) Montréal Canada; ^11^ Department of Physiology and Pharmacology Tel Aviv University School of Medicine Tel Aviv Israel; ^12^ Unit of Metabolic Bone and Thyroid Disorders Fondazione Policlinico Universitario Campus Bio‐Medico and Unit of Endocrinology and Diabetes, Campus Bio‐Medico University Rome Italy; ^13^ Geneva University Hospitals and Faculty of Medicine Geneva Switzerland; ^14^ Division of Endocrinology, Diabetes, Metabolism, and Nutrition and Department of Medicine Mayo Clinic Rochester MN USA; ^15^ Oxford National Institute for Health Research (NIHR) Biomedical Research Centre Oxford University Hospitals NHS Foundation Trust, John Radcliffe Hospital Oxford UK

**Keywords:** PRIMARY HYPERPARATHYROIDISM, PARATHYROID HORMONE, CALCIUM SENSING RECEPTOR, FAMILIAL PHPT, GENETIC TESTS

## Abstract

In this narrative review, we present data gathered over four decades (1980–2020) on the epidemiology, pathophysiology and genetics of primary hyperparathyroidism (PHPT). PHPT is typically a disease of postmenopausal women, but its prevalence and incidence vary globally and depend on a number of factors, the most important being the availability to measure serum calcium and parathyroid hormone levels for screening. In the Western world, the change in presentation to asymptomatic PHPT is likely to occur, over time also, in Eastern regions. The selection of the population to be screened will, of course, affect the epidemiological data (ie, general practice as opposed to tertiary center). Parathyroid hormone has a pivotal role in regulating calcium homeostasis; small changes in extracellular Ca++ concentrations are detected by parathyroid cells, which express calcium‐sensing receptors (CaSRs). Clonally dysregulated overgrowth of one or more parathyroid glands together with reduced expression of CaSRs is the most important pathophysiologic basis of PHPT. The spectrum of skeletal disease reflects different degrees of dysregulated bone remodeling. Intestinal calcium hyperabsorption together with increased bone resorption lead to increased filtered load of calcium that, in addition to other metabolic factors, predispose to the appearance of calcium‐containing kidney stones. A genetic basis of PHPT can be identified in about 10% of all cases. These may occur as a part of multiple endocrine neoplasia syndromes (MEN1–MEN4), or the hyperparathyroidism jaw‐tumor syndrome, or it may be caused by nonsyndromic isolated endocrinopathy, such as familial isolated PHPT and neonatal severe hyperparathyroidism. DNA testing may have value in: confirming the clinical diagnosis in a proband; eg, by distinguishing PHPT from familial hypocalciuric hypercalcemia (FHH). Mutation‐specific carrier testing can be performed on a proband's relatives and identify where the proband is a mutation carrier, ruling out phenocopies that may confound the diagnosis; and potentially prevention via prenatal/preimplantation diagnosis. © 2022 The Authors. *Journal of Bone and Mineral Research* published by Wiley Periodicals LLC on behalf of American Society for Bone and Mineral Research (ASBMR).

## Graphical abstract

The prevalence and incidence of PHPT vary around the world and depend on a number of factors, the most important being availability of serum calcium (and parathyroid hormone) measurement for screening, which in the Western world has shifted its presentation to an asymptomatic disorder. Clonally dysregulated overgrowth of one or more parathyroid glands together with reduced expression of CaSRs is the most important pathophysiologic basis of PHPT, which may also occur as a part of inherited syndromes or as a nonsyndromic isolated endocrinopathy in 10% of cases. Genetic testing has roles in establishing the clinical diagnosis, although its value requires assessments using GRADE^(176)^ methodology, which is recommended for future consideration.

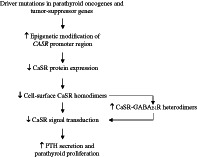

## Introduction

Once considered rare, primary hyperparathyroidism (PHPT) is a common disease of mineral metabolism. The most recent increase in the incidence was driven mainly by routine serum calcium determination or by calcium and parathyroid hormone measurements in the setting of investigations for osteoporosis. The Fourth International Workshop on asymptomatic primary hyperparathyroidism^(^ ^1^ ^)^ did not specifically focus on the global epidemiology of PHPT. Therefore, this narrative review fills this knowledge gap in the context of hypercalcemic PHPT. It is based on the literature review period between 1980 and 2020, utilizing available databases (PubMed, Medline, Embase, Cochrane). This section also highlights recent advances on pathophysiological aspects and genetics of PHPT.

## International Epidemiology of PHPT

### North America

PHPT is a commonly encountered endocrine disorder in North America.^(^ ^2^, ^3^ ^)^ This condition has an equal incidence rate in men and women <45 years of age but is much more common in women after 45 years of age.^(^ ^3^, ^4^ ^)^ The incidence rates of PHPT are highest among blacks, followed by whites with lower rates in Asians and Hispanics.^(^ ^4^ ^)^

Over the past five decades, the epidemiology of PHPT in North America has been highly influenced by changes in medical practice. Prior to 1974, patients were more likely to present with symptomatic PHPT. However, after the introduction of automated chemistry panels in 1974, the incidence rate in Rochester, MN, increased dramatically by identifying patients with asymptomatic PHPT who were previously unrecognized.^(^ ^3^, ^5^ ^)^ By the mid 1980s, the incidence rate of PHPT declined for unclear reasons.^(^ ^3^, ^6^, ^7^ ^)^ In 1998 the incidence rate increased again with current rates of 48.3 to 50.4 per 100,000 person‐years likely due to the introduction of proactive osteoporosis screening guidelines and awareness of new medications for the treatment of osteoporosis.^(^ ^3^, ^4^ ^)^ The most recent estimates on prevalence of PHPT in the United States from 2010 suggest an overall age‐adjusted prevalence rate of 233 per 100,000 in women and 85 per 100,000 in men, with the highest overall prevalence in black and white women aged 70–79 years, with rates of 1409 and 1110 per 100,000 respectively.^(^ ^4^ ^)^

### South America

Individuals in South America with PHPT are mostly symptomatic women, with higher serum calcium levels than those in North America.^(^ ^8^, ^9^ ^)^ Although there are no population‐based studies in South America, data from tertiary care centers suggest that more patients are presenting with asymptomatic PHPT and that this is due to increased availability of serum calcium and parathyroid hormone (PTH) measurements and awareness about PHPT.^(^ ^8^ ^)^

### Western Europe

In Western Europe, as in the United States,^(^ ^3^ ^)^ PHPT is commonly diagnosed in patients with asymptomatic mild hypercalcemia based on multichannel biochemical screening. Regional variability in the use of this screening strategy may explain a higher percentage of diagnosis in symptomatic subjects with renal or skeletal disease and marked hypercalcemia. In addition, more frequent analyses of serum PTH, but not serum calcium, were reported to result in increased detection of PHPT in a Swedish population (*n* = 11,000, 1992–2000).^(^ ^10^ ^)^ This emphasis on PTH measurement is supported by the Parathyroid Epidemiology and Audit Research Study from Scotland, in which intact PTH rather than serum calcium measurement predicted all‐cause mortality and cardiovascular disease.^(^ ^11^ ^)^ The incidence of PHPT has risen across different European countries. In a study from Spain, records from patients with parathyroid disorders (*n* = 12,903, 2003–2017) were obtained; women were 74.7% and admissions due to hyperparathyroidism were 90.23%. The incidence of unspecified hyperparathyroidism increased steadily to 40.3 per 100,000 woman‐years and 13.7 per 100,000 man‐years.^(^ ^12^ ^)^ Women accounted for 90% of all hospital admissions for PHPT. A study in Denmark reported a linear increase in the incidence of PHPT from 1977 to 2010 with an annual rate of 16 per 100,000 in 2010.^(^ ^13^ ^)^ During this period, the incidence was higher in women than in men, with women, but not men, aged ≥50 years having a fivefold increase in incidence. Data from Scandinavian countries report a prevalence of 2%–5% in perimenopausal and postmenopausal women when data are derived from observational and case control studies.^(^ ^14^ ^)^ The prevalence of PHPT derived from Osteoporotic Fractures in Men (MrOs) Sweden has been estimated to be at a much lower rate (0.73) in men than in women.^(^ ^15^ ^)^ A retrospective Italian analysis (2006–2011) of 46 ,275 hospitalizations for episodes of PHPT identified a female predominance of 69% of patients with PHPT.^(^ ^16^ ^)^ Consistently, between 2000 and 2010, three times as many women as men underwent parathyroidectomy for PHPT in England and Wales.^(^ ^17^ ^)^

### Eastern Europe

Currently, there are no published studies specifically for the incidence or prevalence of PHPT in the general population of Eastern European countries. However, incidence and prevalence data are mentioned in some publications that are assumed to be from official national statistics. Thus, data from the Czech Republic indicate an incidence of PHPT of 24 cases per 100,000 persons per year,^(^ ^18^ ^)^ and the prevalence of PHPT in Serbia is estimated as 0.3% in the general population and 1.89% in the population referred for investigation of thyroid and parathyroid disorders.^(^ ^19^ ^)^ PHPT is more common among women than men, and in the elderly population.^(^ ^18^, ^20^, ^21^ ^)^ PHPT prevalence is much higher in patients with low bone mass (11.5% in Poland),^(^ ^22^ ^)^ urolithiasis (3.72% of confirmed PHPT in Russia),^(^ ^23^, ^24^ ^)^ and among patients undergoing thyroid surgery (10.1% in Poland).^(^ ^25^ ^)^ From a total of 2662 thyroid ultrasound scans in Romania, 32 patients were identified with parathyroid incidentaloma and PHPT was confirmed in 12 patients.^(^ ^26^ ^)^

The prevalence of hereditary causes was reported at 10.6% among patients with PHPT in a single Hungarian center^(^ ^27^ ^)^ and 14% in Serbian patients younger than 19 years.^(^ ^28^ ^)^ Parathyroid cancer was found in 0.19% of PHPT patients in the Czech Republic^(^ ^29^ ^)^ and in 2.1% of PHPT patients in a Latvian center.^(^ ^30^ ^)^

### Asia, Australia, and Africa

PHPT is underdiagnosed in the developing world mainly because of a lack of routine serum biochemical screening. Not surprisingly, it presents as a more severe disease with a greater proportion of patients presenting with classical bone and renal impairment.^(^ ^31^, ^32^ ^)^ PHPT in developing countries predominantly affects women at younger ages and with renal and musculoskeletal involvement because of concomitant vitamin D insufficiency.^(^ ^31^ ^)^ However, PHPT in China during the past 15 years is evolving into a more asymptomatic condition due to earlier detection of hypercalcemia by increased availability of multichannel autoanalyzers and the use of routine neck ultrasonography that has increased the finding of incidental parathyroid lesions.^(^ ^33^, ^34^, ^35^, ^36^ ^)^ Prevalence of PHPT in middle‐aged and elderly Chinese (*n* = 2451) was reported to be 0.2%.^(^ ^37^ ^)^ A single‐center retrospective study (2013–2016) estimated PHPT occurrence as 0.4% in patients hospitalized for urolithiasis in Korea.^(^ ^38^ ^)^ PHPT epidemiological data within the last two decades have not been reported from Japan or in India, where health policy does not include screening of asymptomatic subjects with serum calcium, but where PHPT remains a severe disease (http://www.indianphptregistry.com).^(^ ^39^, ^40^, ^41^ ^)^

Retrospective studies of PHPT in Australia have reported marked increases in age‐standardized rates of parathyroidectomy for PHPT in women from 0.14 cases per 100,000 in 1976 to 7.7 cases per 100,000 in 1996 (total parathyroidectomies = 1506, University of Sydney); and in the rates of parathyroidectomy in New South Wales (women from 5.1 cases per 100,000 in 1993 to 12.3 cases per 100,000 in 1998, and men from 2.1 per 100,000 in 1993 to 4.7 per 100,000 in 1998). Osteoporosis, which occurred in 34% of patients, replaced kidney stones as the most common indication for surgery. Mortality was reported to be significantly (*p* < 0.001) greater in PHPT patients (*n* = 561, from Sydney) when compared to that of the Australian population studied during the same time interval (1961–1994)^(^ ^42^ ^)^; PHPT epidemiological data within the last two decades have not been reported from New Zealand.

Information about PHPT epidemiology and clinical presentation in Africa^(^ ^31^ ^)^ are derived mainly from case reports, small case series, and retrospective evaluations that are principally from South Africa. A single‐center (Cape Town) prospective hospital in‐patient (*n* = 58,053, 1983–84) study reported that 0.6% patients had hypercalcemia, and that 16.5% of these patients had PHPT (incidence = 78 per 100,000 years hospital in‐patients) which represented the second most common cause of hypercalcemia.^(^ ^43^ ^)^ Other single‐center hospital inpatient studies have reported a PHPT prevalence of 21.3% in hypercalcemic patients (*n* = 560, from Johannesburg),^(^ ^44^ ^)^ with >90% and ~80% patients (*n* = 28, from Durban) being symptomatic and females, respectively, and the mean age at presentation being 60 years.^(^ ^45^ ^)^ Table 1 summarizes the reported prevalence and incidence in the world.

**Table 1:** Incidence and Prevalence of Primary Hyperparathyroidism in the World

Area	Incidence per 100,000 person‐years	Prevalence per 100,000 people	
USA	48.3–50.4	Women	233
		Men	85
		Black women, 70–79 years old	1409
		White women, 70–79 years old	1110
Spain	Unspecified type of hyperparathyroidism		
Women	40.3		
Men	13.7		
Denmark	16		
Scandinavian countries		Observational studies in perimenopausal and postmenopausal women	2000–5000
Sweden		Men	730
Czech Republic	24		
Serbia		General population	300
China		Middle‐aged and elderly	200
South Africa (hospital inpatients)	78		

## Pathophysiological Aspects of PHPT

### PTH physiology

PTH plays a central role in the regulation of calcium homeostasis^(^ ^46^ ^)^ (Table 2). Small alterations in the extracellular ionized calcium concentration (Ca^++^) are detected by a cell membrane‐associated calcium‐sensing receptor (CaSR), which also recognizes other divalent cations.^(^ ^47^ ^)^ However, Ca^++^ is the most sensitive ligand. Activation of the CaSR inhibits PTH secretion, PTH gene expression and parathyroid cell proliferation. CaSR functional insufficiency attenuates the suppression of PTH release by increased extracellular Ca^++^; ie, the PTH secretion versus Ca^++^ curve is shifted to the right.^(^ ^47^ ^)^ Phosphate appears to interact with the CaSR as well.^(^ ^48^ ^)^ Calcitriol (1,25‐dihydroxyvitamin D) reduces PTH gene expression and parathyroid cells proliferation through the vitamin D receptor, which is also expressed in parathyroid cells.^(^ ^49^ ^)^

**Table 2:** PTH Actions and Their Pathophysiology

Target organ	PTH cell target	PTH‐regulated function	Pathophysiological changes	Clinical implications
Kidney	Distal tubule	Calcium reabsorption	Hypercalcemia (with contributions from gut and bone)	Hypercalcemic syndrome Increased mortality
	Proximal and distal tubules	Phosphate reabsorption	Hypophosphatemia	Fatigue/muscle weakness
	Distal tubule	Bicarbonate reabsorption	Hyperchloremic acidosis	Nephrocalcinosis
	Proximal tubule	1‐Alpha hydroxylase	Hypercalciuria (indirectly)	Renal stones
Gut	Proximal and distal intestine	Indirect through 1,25OH2D‐dependent Increased calcium intestinal absorption	Hypercalciuria	Renal stones
Bone	Osteoblast	Bone turnover	High bone turnover Bone loss	Fracture
Cardiovascular system	Cardiomyocyte	Hypercalcemia‐dependent	Arrythmias	Possible increase in mortality
		Interaction with RAAS	Left ventricular hypertrophy	
			Heart failure	Heart failure
			Soft tissue calcification	
	Cardiac valves	Hypercalcemia‐dependent	Soft tissue calcification	
	Smooth muscle cells	Vasodilatation	Decreased blood pressure	Decreased blood pressure
		Interaction with RAAS	Hypertension*	Hypertension
			Soft tissue calcification	
Central nervous system		Hypercalcemia	Apoptosis*	
	Axons	Cross‐reactivity with PTH2R	Stress response, anxiety*	
Skeletal muscle	Myotube		Muscle weakness	
Dermis	Fibroblasts/hair follicles	Possible role hair growth/differentiation	None known in nongenetic forms of PHPT	No known

* Possible.

In the kidney, the amount of calcium excreted represents the difference between filtered load and net tubular reabsorption. The latter is the key determinant of Ca^++^ extracellular concentration and homeostasis.^(^ ^46^ ^)^ Twenty percent to 30% of filtered calcium is reabsorbed along the ascending limb of the renal tubule whereas 10% is reabsorbed in the distal tubule in response to PTH^(^ ^46^ ^)^ (Table 2). PTH also stimulates the renal conversion of 25‐hydroxyvitamin D to 1,25‐dihydroxyvitamin D, which in turn increases intestinal calcium and phosphate absorption.^(^ ^49^ ^)^ Approximately 70% of the filtered inorganic phosphate is reabsorbed in the proximal tubule through saturable sodium‐phosphate co‐transporters (NaPTs). PTH stimulates the removal of NaPi2a and NaPi2c from the brush border membrane.^(^ ^50^ ^)^ This action of PTH, combined with cellular degradation of these transporters, results in renal phosphate wasting and hypophosphatemia. In the distal tubule, PTH reduces bicarbonate reabsorption, accounting for the state of mild hyperchloremic acidosis observed in some cases of PHPT.^(^ ^51^ ^)^

In adults, net intestinal absorption of calcium constitutes approximately 20% of ingested calcium. The intestinal calcium absorptive capacity is mainly controlled by calcitriol, which stimulates calcium transport through both genomic and nongenomic mechanisms.^(^ ^52^ ^)^ PTH only indirectly affects intestinal calcium and phosphate uptake via its actions on vitamin D metabolism^(^ ^53^ ^)^ (Table 2). Transapical membrane transport of calcium through the transient receptor potential vanilloid subfamily member 6 (TRPV6) channel is stimulated by calcitriol, whereas the extrusion at the basolateral membrane is carried out by the plasma‐membrane calcium pump isoform 1 (PMCA1), also known as ATPase Plasma Membrane Ca2+ Transporting 1 (ATP2B1).^(^ ^52^ ^)^ Calcitriol may also regulate paracellular calcium transport by acting on various tight junction proteins. The duodenum is most sensitive to the stimulatory effect of calcitriol on calcium absorption. Colonic mucosa also has a vitamin D–sensitive calcium transport mechanism but little calcium is absorbed in the colon because, at that point, calcium is largely complexed to various anions.^(^ ^54^ ^)^ Metabolism of prebiotics by gut microbiota decreases large intestinal content pH variation and increases calcium absorption.^(^ ^55^ ^)^ At steady state, 24‐hour urinary calcium excretion mainly reflects daily net intestinal calcium absorption and net bone resorption.

On average, about 1% of total bone calcium exchanges every month, through a mechanism involving bidirectional fluxes mediated by the bone remodeling cycle.^(^ ^46^ ^)^ The main regulators of these fluxes are PTH and calcitriol. In the absence of PTH, bone turnover is very low. It appears that PTH requires elements of gut microbiota to stimulate both bone resorption and bone formation.^(^ ^56^ ^)^

Cardiomyocytes and vascular smooth muscle cells express the PTH receptor (PTHR).^(^ ^57^ ^)^ PTH exerts inotropic and chronotropic actions on adult cardiomyocytes, whereas peripherally it relaxes vascular smooth muscle cells and causes vasodilatation. The dynamic between these cardiac and peripheral vascular effects of PTH may provide an explanation for the occurrence of hypertension or hypotension that is observed in associations with chronic excess of PTH in some patients.^(^ ^57^, ^58^ ^)^ Another possible mechanism for hypertension may also be the cross‐talk between PTH signaling and the renin‐angiotensin‐aldosterone system^(^ ^57^ ^)^ (Table 2). PTH activation of protein kinase C, leading to hypertrophic growth, may account for left ventricular hypertrophy that is sometimes observed with PTH excess.^(^ ^57^ ^)^

Administration of PTH (1‐34) to rats induces calcium uptake in brain synaptosomes independent of protein kinase A (PKA) activation.^(^ ^59^ ^)^ PTHR1 and PTHR2 transcripts are detectable in various brain regions.^(^ ^60^ ^)^ PTHR2 appears to affect neural and neuroendocrine functions, including the stress response, thermoregulation, and prolactin release.^(^ ^61^ ^)^ Blocking PTHR2 signaling is associated with a higher stress state in experimental animals.

### Physiological roles of the parathyroid and kidney CaSR

The CaSR is a homodimeric family C G‐protein‐coupled receptor (GPCR) that is most highly expressed in the parathyroid glands. There it influences systemic Ca^2+^ homeostasis by detecting increases in the prevailing circulating Ca^2+^ concentration, which leads to an acute decrease in PTH secretion.^(^ ^62^, ^63^ ^)^ The CaSR couples to heterotrimeric G proteins of the G_q/11_ and G_i/o_ classes, which mediate signaling via intracellular Ca^2+^ mobilization and the mitogen‐activated protein kinase (MAPK) cascade.^(^ ^64^ ^)^ The adaptor protein 2 (AP2) complex increases CaSR endocytosis and promotes CaSR signaling within endosomes.^(^ ^65^ ^)^ In the parathyroid, the CaSR also induces synthesis of 1,25‐dihydroxyvitamin D, which may contribute to the autocrine/paracrine suppression of PTH secretion.^(^ ^66^ ^)^ In addition, the CaSR regulates parathyroid cell proliferation potentially via the MAPK pathway,^(^ ^67^ ^)^ and in part by an interaction with the parathyroid‐expressed klotho protein.^(^ ^68^ ^)^ In the kidney, the CaSR is most highly expressed in the renal thick ascending limb of the Loop of Henle, where it inhibits paracellular reabsorption of Ca^2+^ mediated by claudin proteins.^(^ ^64^ ^)^ The CaSR regulates Ca^2+^ reabsorption likely by inhibiting expression of microRNA (miR) molecules, miR‐9 and miR‐374, which leads to increased claudin‐14 expression.^(^ ^69^ ^)^

### Clinical consequences of dysregulated parathyroid function

More details about the clinical features of classical PHPT and other aspects of the disease are found in the accompanying article.^(69)^ The cardinal biochemical finding in PHPT is hypercalcemia (Table 2). Clonally dysregulated overgrowth of one or more parathyroid glands, accompanied by a reduced expression of the CaSR in that tissue, is the most common pathophysiologic basis for this finding.^(^ ^70^, ^71^ ^)^ Thus, there is both a mass effect, with a net increase in the amount of PTH being secreted as well as an altered set‐point for calcium‐mediated suppression of PTH secretion. Perhaps because of these dual pathophysiologic mechanisms, serum levels of PTH do not correlate particularly well with the size of the adenoma in typical cases of PHPT^(^ ^72^, ^73^ ^)^ and even when such a correlation has been reported, gland size has been found to vary widely with a given level of circulating PTH, especially when the PTH value is mildly to moderately elevated.^(^ ^74^ ^)^ Extreme elevations in PTH do, however, raise the specter of parathyroid carcinoma.

The source of the increase in serum Ca^++^ varies depending on the severity of the hypercalcemia. In mild‐to‐moderate disease, both an increase in bone resorption and postprandial calcitriol‐mediated intestinal calcium hyperabsorption contribute to the hypercalcemia^(^ ^75^, ^76^ ^)^ (Table 2). Intestinal calcium transport, which largely takes place in the proximal intestine, occurs by both transcellular and paracellular pathways. The transcellular pathway is a tightly regulated pathway and is increased by 1,25(OH)_2_ vitamin D. The paracellular pathway can also be regulated by modulation of tight junction proteins such as claudin 2 and claudin12. Expression of both claudins is increased by 1,25(OH)_2_ vitamin D thereby augmenting paracellular calcium transport.^(^ ^77^ ^)^ The increase in distal tubular calcium reabsorption plays a greater role in sustaining the hypercalcemia than intestinal calcium absorption. In the typical patient with mild to moderate PHPT, plasma calcium and PTH levels can remain stable for years and when the hypercalcemia is mild (ie, less than 11.0 mg/dL [2.75 mmol/L]) patients often have relatively few symptoms. When the hypercalcemia is more severe, nausea, vomiting, dehydration, muscle weakness, and impaired mentation can occur. Rarely a patient may experience sudden worsening of PHPT, so called parathyroid storm or acute hyperparathyroidism.^(^ ^78^, ^79^ ^)^ Although PTH lowers the renal phosphate threshold, frank hypophosphatemia is uncommon in most cases of PHPT. Low‐normal phosphate values are commonly seen in PHPT, although it is not as reliable a finding as hypercalcemia. Fibroblast growth factor 23 (FGF23) is elevated in patients with PHPT and correlates positively with serum calcium and PTH and negatively with levels of serum phosphorus and 1,25(OH)_2_ vitamin D.^(^ ^80^ ^)^ However, in a multiple regression analysis only serum calcium and creatinine clearance were predictors of FGF23, and serum levels of FGF23 did not change after curative surgery, suggesting that this hormone is not likely to play a major role in mediating the hypophosphatemia seen in PHPT.^(^ ^80^ ^)^ Vitamin D deficiency is also frequently seen in PHPT and can exacerbate its severity.^(^ ^81^, ^82^ ^)^

Because, as just noted, the more well‐established consequences of dysregulated parathyroid function are covered elsewhere in this series, the remainder of this section is devoted to selected new and emerging aspects of the pathophysiology of PHPT as well as to areas of uncertainty.

#### The role of estrogen deficiency in PHPT

Following menopause, the efficiency of intestinal calcium absorption declines and continues through the later postmenopausal years.^(^ ^83^, ^84^ ^)^ Estrogen has many trophic effects on the skeleton. Estrogen deficiency results in increased osteocyte apoptosis.^(^ ^85^ ^)^ In addition, estrogen directly induces osteoclast apoptosis,^(^ ^85^ ^)^ a restraining effect on bone resorption that is lost following menopause. This plays a key pathogenic role in the accelerated loss of bone that occurs in the early postmenopausal years. The loss of the inhibitory effect of estrogen on bone resorption sensitizes the skeleton to the resorptive effects of excess PTH in PHPT and may partially explain in increased incidence of PHPT in postmenopausal women.

#### Environmental chemicals and PHPT

The incidence of PHPT increases with age in both men and women. There are likely many factors that contribute to this. One that has received recent attention is the role of environmental chemicals. Rats fed polychlorinated biphenyls (PCBs) develop secondary hyperparathyroidism due, in part, to increased metabolism of vitamin D.^(^ ^86^ ^)^ Hu and colleagues^(^ ^87^ ^)^ reported the presence of environmental chemicals in the majority of parathyroid tissue from patients with secondary and primary hyperparathyroidism. Many of these environmental chemicals are considered to be endocrine disrupters. Interestingly, tissue content of PCB‐28 and PCB‐49 correlated positively with parathyroid tissue mass although this was primarily driven by the data from patients with secondary hyperparathyroidism due to renal failure.^(^ ^87^ ^)^

#### Gender bias in PHPT

Although data are limited, available evidence does not suggest gender bias in the frequency with which patients with PHPT are referred for surgery or in surgical outcomes.^(^ ^88^, ^89^ ^)^

#### Obesity and PHPT

Severe obesity has long been recognized as a risk factor for hypovitaminosis D and can be associated with secondary hyperparathyroidism^(^ ^90^ ^)^; studies also suggest a relationship between obesity and PHPT. Grey and colleagues^(^ ^91^ ^)^ and Grey and Reid^(^ ^92^ ^)^ found that postmenopausal women with PHPT were heavier and had increased body fat in an android distribution compared to age‐matched controls with normal parathyroid function. Further, premenopausal obesity appeared to precede the development of PHPT. Adam and colleagues^(^ ^93^ ^)^ reported that severely obese patients with PHPT had higher serum levels of PTH and had larger adenomas than non‐obese patients. This relationship was independent of vitamin D levels. However, some studies indicate that elevated PTH levels are associated with reduced body weight.^(^ ^94^ ^)^ Obese PHPT patients have been found to have a higher incidence of hypercalciuria and nephrolithiasis but to be less prone to low bone mass.^(^ ^95^ ^)^

#### The renin/angiotensin/aldosterone axis in PHPT

Epidemiologic studies report an increased incidence of cardiovascular disease, including left ventricular hypertrophy, stroke, and hypertension.^(^ ^57^ ^)^ Left ventricular hypertrophy may regress after parathyroidectomy.^(^ ^96^ ^)^ The effects of surgical cure of PHPT on hypertension is unclear. Given this ongoing controversy, the relationship between the renin/angiotensin/aldosterone axis and PHPT has been extensively studied. Both acute and chronic effects of PTH itself as well as of calcium on this axis have been reported.^(^ ^97^, ^98^ ^)^ Some investigators have found elevated plasma levels of angiotensin in patients with PHPT.^(^ ^99^ ^)^ However earlier work found no effect of parathyroidectomy on the plasma renin activiy (PRA) axis in patients with PHPT.^(^ ^100^, ^101^ ^)^ Further, elevated levels of 1,25vitamin D often seen in PHPT would, based on recent data, suppress the PRA axis.^(^ ^102^ ^)^

#### Central nervous system effects of PHPT

The neuropsychiatric symptoms seen in PHPT include depression, anxiety, memory loss, and difficulty with concentration.^(^ ^103^ ^)^ Because both PTH receptors and the CaSR are expressed in the central nervous system (CNS), these symptoms could reflect PTH excess. Consistent with this idea, concentrations of PTH are higher in the cerebral spinal fluid of patients with HPT.^(^ ^104^ ^)^ However, the efficacy of treatment in ameliorating these symptoms remains unclear.^(^ ^105^ ^)^

#### Renal calcium leak

Very rarely, despite successful surgical treatment and normal to low‐normal postoperative serum calcium levels, hypercalciuria persists in patients with PHPT. In those cases, it is possible that the underlying pathophysiology was actually a primary renal calcium leak^(^ ^106^, ^107^ ^)^ that led to secondary and eventually surfaced clinically as PHPT.

#### PTH and skin structures

The PTH receptor is expressed in human dermal fibroblasts and is responsive to PTH in vitro.^(^ ^108^ ^)^ The hormone PTH‐related protein (PTHRP) is made by human keratinocytes.^(^ ^109^ ^)^ Although these findings suggest a paracrine role for PTHRP in the dermis, there are no clinically relevant skin findings in patients with sporadic, nongenetic forms of PHPT. PTH receptors are also expressed by hair follicles^(^ ^110^ ^)^ but again there are no notable changes in hair growth or cycling in PHPT.

Table 2 presents a summary of organ systems that are in fact, or potentially could be, targets in PHPT. The table summarizes the effects of PTH with regard to its cellular, pathophysiological, and clinical consequences. A detailed discussion of these points as they relate to clinical consequences is provided in an accompanying report by El‐Hajj Fuleihan and colleagues in this series (see accommpanying paper of Task Force 6 by El‐Hajj Fuleihan G. et al.).

### Genetics of PHPT

A genetic basis for PHPT occurs in about 10% of all patients with PHPT. These forms of PHPT (Table 3) may occur as part of the complex syndromes (eg, multiple endocrine neoplasia [MEN] types 1, 2A, and 4 [MEN1, MEN2A, and MEN4]; and the hyperparathyroidism‐jaw tumor [HPT‐JT] syndrome); or a nonsyndromic isolated endocrinopathy such as familial isolated PHPT (FIHP), neonatal severe hyperparathyroidism (NSHPT), or familial hypocalciuric hypercalcemia (FHH). In general, DNA testing can be impactful by: confirming the clinical diagnosis in a proband; determining if mutation‐specific carrier testing can be offered to a proband's relatives, which requires a detectable mutation in the proband; determining whether asymptomatic or other relatives of a proband are mutation carriers; ruling out phenocopies that may confound the diagnosis; and potentially, prevention via prenatal/preimplantation diagnosis. The specific clinical impact of this testing varies among the PHPT disorders.

**Table 3:** Familial Primary Hyperparathyroidism—Major Genetic Basis and Key Features

Clinical diagnosis	Major gene/protein	Distinguishing aspects of hyperparathyroidism	Additional features/considerations
FHH		Autosomal dominant	Low CCCR
FHH1	*CASR/*CaSR^a^	Lifelong hypercalcemia (otherwise rare in first decade of life in other syndromes)	Often increased serum Mg
FHH2	*GNA11*/Gα11	PTH levels inappropriately normal or mildly elevated	No increase in nephrocalcinosis or stones Normal bone mass No or minimal symptoms of hypercalcemia suggesting decreased sensitivity in other tissues expressing CASR eg, brain, GI tract Antibodies against CaSR can give similar picture, but usually with other autoimmune features and nonfamilial Heterozygosity for the defective CASR, GNA11, or AP2S1 allele
FHH3	*AP2S1*/adaptor‐protein 2 σ‐subunit	Calcium‐PTH setpoint curve shifted to right = decreased sensitivity of PTH release to suppression by ambient calcium Normal or mildly hypercellular parathyroid glands Persistent hypercalcemia after subtotal PTX. PTX to be generally avoided Rare individuals/kindreds with *CASR* mutation have phenotype on spectrum to typical sporadic PHPT, can include hypercalciuria and benefit from PTX	
NSHPT	*CASR*/CaSR	Autosomal recessive or dominant Severe hypercalcemia begins at birth; very high PTH levels Parathyroid glands all exceedingly large, hypercellular Requires urgent total PTX	Fractures, hypotonia, respiratory distress; neurodevelopmental impairment if survives without early treatment Low CCCR Compound heterozygosity or homozygosity for inactive CASR alleles ‐ parental consanguinity as one cause of latter
MEN1	*MEN1*/menin CDK inhibitor genes (other than *CDKN1B*) in rare families: *CDKN1A*, *CDKN2B*, *CDKN2C*/p21, p15, p18	Autosomal dominant Multigland parathyroid disease; gland size asymmetry but all glands hypercellular; avoid minimally invasive PTX Onset often in second/third decade	Enteropancreatic endocrine tumors with malignant potential are main life‐threatening feature; also tumors of anterior pituitary, adrenal, skin, and bronchial/thymic carcinoids, lipomas, others
MEN4	*CDKN1B*/p27	High recurrence rate years after successful subtotal PTX Thymus as common location for pathologic parathyroid tissue upon recurrence of HPT; thymectomy advised on initial PTX	
MEN2A	*RET*	Autosomal dominant Generally mild multigland disease, less likely to recur after subtotal PTX than in MEN1	Medullary thyroid carcinoma Pheochromocytoma RET DNA testing enables lifesaving prophylactic thyroidectomy
HPT‐JT	*CDC73* (*HRPT2*)	Autosomal dominant Hypercalcemia at times in first decade, usually later All glands at risk of neoplasia, which may develop asynchronously Mostly benign adenomas and atypical adenomas, with marked increased risk of parathyroid cancer (15%) Evidence for evolution from benign to malignant neoplasia At times cystic or microcystic histopathology Tumors may grow rapidly Germline mutations in subset of patients with sporadic parathyroid carcinoma Close surveillance of normocalcemic mutation‐positive carriers to enable early curative surgery Reports of poorly functioning parathyroid carcinomas Bilateral exploration advised for PTX: resection of abnormal glands; avoid prophylactic total PTX	Benign ossifying fibromas of mandible and maxilla, renal cysts, uterine tumors
FIHP	*CASR*, *MEN1*, or *CDC73* in 30% Additional causes/contributors pending investigation/confirmation^b^	Genetic heterogeneity, different modes of inheritance, different patterns of parathyroid pathology	No other clinical features (as per definition) Emergence of a syndromic feature redefines patient/kindred out of this category

CCCR = calcium to creatinine clearance ratio; PTX = parathyroidectomy.

^a^ Germline *CASR* mutations are also a rare cause of sporadic primary hyperparathyroidism.

^b^ Includes candidate *GCM2* variants of uncertain penetrance, with enhanced transcriptional activity in vitro, with role in clinical management not yet established; in addition, PHPT may be a rare feature associated with germline mutations in MAX (in a familial pheochromocytoma/paraganglioma syndrome tentatively designated “MEN5”) and SLC12A1 (see main text, Genetics of PHPT).

### Syndromic forms of PHPT

#### MEN1

MEN1 is characterized by the occurrence of PHPT in association with enteropancreatic neuroendocrine tumors (NETs), anterior pituitary tumors, adrenocortical tumors, bronchial/thymic carcinoids, lipomas, and other skin lesions^(^ ^111^ ^)^ (Table 3). Notably, PHPT is a very penetrant, multigland disease, highly prone to recurrence even after apparently successful subtotal parathyroidectomy.^(^ ^112^, ^113^ ^)^ Heterozygous inactivating germline mutation in *MEN1*, a tumor suppressor gene encoding menin, is the major genetic basis and is detectable in ≥70% of classically affected kindreds.^(^ ^111^, ^114^ ^)^ However, the responsible mutation in rare kindreds with a clinical diagnosis of MEN1 instead lies in *CDKN1B*, encoding the p27 cyclin‐dependent kinase inhibitor (CDKI)^(^ ^115^ ^)^; the term MEN4 has been applied to this setting (Table 3). Still other individuals/kindreds with an MEN1 phenotype carry missense variants in a different CDKI gene—either *CDKN1A*, *CDKN2B*, or *CDKN2C* ^(^ ^116^ ^)^; reports of these remain quite limited, as is penetrance information. Germline mutations in *MEN1* are reported in a some kindreds with FIHP; such mutations or those in the stated CDKI genes have rarely been uncovered in patients with sporadic PHPT, the former skewed to younger ages.^(^ ^117^, ^118^, ^119^ ^)^

In contrast to the high efficacy and impact of DNA testing established for analysis of the *rearranged during transfection* (*RET)* proto‐oncogene for MEN2A/2B (see section MEN2A below), the quality of the evidence base supporting DNA diagnosis for MEN1/4 is less robust; eg, due to a dearth of randomized or well‐controlled studies demonstrating improved morbidity or mortality compared with clinical diagnosis. Still, based on expert opinion/experience, there are multiple situations in which DNA testing can alter management to the benefit of patient and/or family.^(^ ^111^, ^120^ ^)^ Examples can include: establishing a genetic diagnosis of MEN1 in a patient with a suggestive/questionable family history presenting with isolated PHPT; in sporadically presenting Zollinger‐Ellison syndrome, or patients undergoing parathyroidectomy, where revealing MEN1 can alter the approach to treatment; in a MEN1 proband in order to enable definitive testing of asymptomatic relatives; in clinically unaffected members of a MEN1 kindred, to appropriately engage surveillance for early detection of tumors in mutation carriers, and to eliminate anxiety and costs of surveillance via definitively excluding carrier status; and in detecting MEN1 phenocopies.^(^ ^121^ ^)^

#### MEN2A

MEN2A is characterized by the occurrence of PHPT in association with medullary thyroid carcinoma (MTC) and pheochromocytoma (Table 3).^(^ ^122^, ^123^ ^)^ PHPT manifests with a lower penetrance and usually later than MTC and pheochromocytoma.^(^ ^124^ ^)^ Gain‐of‐function *RET* mutations cause MEN2A (Table 3) and are readily detected by DNA testing, but the diagnosis is generally apparent by the time PHPT presents.^(^ ^123^, ^124^, ^125^ ^)^

#### Hyperparathyroidism‐jaw tumor syndrome

Hyperparathyroidism‐jaw tumor syndrome (HPT‐JT) is characterized by the occurrence of parathyroid tumors, which are mostly benign adenomas and atypical adenomas although ~15% may be malignant, in association with ossifying fibromas of the jaw and benign and malignant tumors of the kidneys and uterus^(^ ^126^, ^127^ ^)^ (Table 3). PHPT is highly penetrant, with all parathyroids at risk for tumor development in an often‐asynchronous manner, and with a substantially increased risk of atypical adenomas and parathyroid malignancy. Heterozygous inactivating germline mutation of the *cell division cycle 73* (*CDC73*) tumor suppressor gene encoding parafibromin, is the major genetic abnormality and is detectable in about 70% of classically affected kindreds.^(^ ^128^, ^129^ ^)^ *CDC73* mutations can also be found in 5%–10% of probands presenting with FIHP^(^ ^125^ ^)^ and, importantly, in 20%–30% of patients with sporadically‐presenting parathyroid carcinoma.^(^ ^130^, ^131^, ^132^ ^)^

DNA testing for *CDC73* mutations has been quite beneficial in clinical practice, based on expert opinion and published reports, although it must be acknowledged that high‐quality evidence of improved outcomes; eg, from randomized studies, are lacking—in large part due to the rarity of HPT‐JT and parathyroid carcinoma. The major benefit is to identify clinically unaffected mutation‐positive carriers for surveillance and monitoring, with the aim of early diagnosis/treatment of PHPT to cure or prevent malignancy and avoid premature death.^(^ ^133^, ^134^ ^)^ Thus, DNA testing can alter management in situations including: establishing a genetic diagnosis of HPT‐JT in a patient with isolated PHPT and a suggestive/questionable family history that may include parathyroid carcinoma; in sporadically presenting parathyroid carcinoma, where revealing a germline *CDC73* mutation can alter the approach to surgery and family surveillance; in a HPT‐JT proband in order to enable definitive testing of asymptomatic relatives; in clinically unaffected members of a HPT‐JT kindred, to appropriately engage surveillance for early detection of tumors, and to eliminate anxiety and costs of surveillance by excluding mutation carrier status.^(^ ^135^, ^136^, ^137^, ^138^ ^)^

#### Other genetic considerations for syndromic PHPT

Germline mutations in the *MAX* tumor suppressor gene have been strongly associated with a syndrome of familial pheochromocytoma/paraganglioma,^(^ ^139^ ^)^ and a few affected individuals in such kindreds have also been reported to have PHPT, mostly without a clear pathologic or pathophysiologic basis.^(^ ^139^, ^140^, ^141^ ^)^ At this time the potential inclusion of PHPT in the definition of this syndrome is intriguing but evidence (genetic and clinicopathologic) is extremely limited and further study is needed. Similarly, additional evidence is needed to understand and better define the apparently rare development of biochemical PHPT in neonates and children with germline *SLC12A1* mutation.^(^ ^142^, ^143^ ^)^

### Nonsyndromic forms of PHPT

#### FIHP

FIHP is genetically heterogeneous, with about 30% of such kindreds carrying germline *MEN1* or *CDC73* mutations with incomplete expression, or loss‐of‐function mutations of the *CASR* gene (Table 3).^(^ ^125^, ^144^, ^145^, ^146^ ^)^ The genetic basis for most FIHP kindreds remains unknown.

Specific variants of the *glial cells missing 2* (*GCM2*) gene, which encodes a transcription factor, have been proposed to cause a subset of FIHP,^(^ ^147^, ^148^ ^)^ but important questions remain unanswered and this should be considered an interesting candidate pending further study. In contrast to established causes of FIHP such as inactivated alleles of *MEN1* or *CDC73* (Table 3), the main *GCM2* variants are found at much higher‐order frequencies in the general population^(^ ^148^, ^149^ ^)^ with very limited data on their penetrance and with functional evidence only from in vitro transcriptional activity that heretofore is unlinked to the PHPT phenotype. Intriguingly, these variants do appear to be overrepresented in cohorts of familial and sporadic PHPT,^(^ ^148^, ^149^ ^)^ some with relatively pronounced manifestations,^(^ ^150^ ^)^ and their potential contribution should be actively investigated, including the possibility that they could be genetic modifiers.

#### FHH and PHPT caused by germline mutations of the CaSR and partner proteins

FHH is an autosomal dominant disorder characterized by lifelong elevations of serum calcium concentrations, mild hypermagnesemia, normal or mildly raised serum PTH concentrations, and low urinary calcium excretion (Table 3).^(^ ^64^ ^)^ Most affected individuals have minimal or no symptoms, and no adverse consequences to their bones or other end‐organs. There are three genetic types of FHH (FHH1–FHH3), which are caused by heterozygous loss‐of‐function mutations of the *CASR* gene, *G‐protein subunit alpha 11* (*GNA11*) and *AP2 sigma‐1* (*AP2S1*) genes, respectively (Table 3).^(^ ^151^ ^)^ FHH1 is the major type with an estimated genetic prevalence of 74.1 per 100,000,^(^ ^152^ ^)^ and is generally asymptomatic, although some patients have been reported to have features such as chondrocalcinosis and osteoporosis.^(^ ^153^ ^)^ Nephrolithiasis also affects some FHH1 patients.^(^ ^153^ ^)^ However, the composition of these renal stones and their underlying pathogenesis remain to be elucidated. FHH2 is the rarest type and reported in four probands to‐date.^(^ ^64^ ^)^ FHH2 is associated with a mild clinical presentation and serum adjusted‐calcium concentrations are usually <2.80 mmol/L (normal range, 2.10–2.55 mmol/L),^(^ ^64^ ^)^ and urinary calcium excretion may be normal or low. FHH3 has an estimated prevalence of 7.8 per 100,000,^(^ ^154^ ^)^ and is associated with more severe hypercalcemia than FHH1.^(^ ^155^, ^156^ ^)^ FHH3 patients may also have low bone mineral density (BMD), osteomalacia, or neurodevelopmental disorders.^(^ ^155^, ^157^ ^)^ Because the underlying genetic abnormality in FHH directly causes alteration in the calcium‐PTH setpoint in all parathyroid cells, surgical excision of parathyroid glands is generally inadvisable and will typically result in persistent hypercalcemia or, if total parathyroidectomy is performed, in the adverse outcome of hypoparathyroidism.

Germline *CASR* mutations can also cause more consequential PHPT disorders. Thus, offspring of parents with FHH1 can harbor biallelic loss‐of‐function *CASR* mutations that cause NSHPT, which is associated with marked hyperparathyroidism that leads to hypercalcemia and bone demineralization, causing fractures and respiratory distress, and generally requires urgent total parathyroidectomy (Table 3).^(^ ^158^ ^)^ A child harboring a monoallelic loss‐of‐function *CASR* mutation is at risk of transient neonatal hyperparathyroidism if born to a normocalcemic mother.^(^ ^159^ ^)^ Loss‐of‐function *CASR* mutations, either heterozygous or homozygous, are occasionally also reported in patients presenting in adulthood with features of typical PHPT such as raised serum PTH, hypercalciuric nephrolithiasis, and/or osteoporosis.^(^ ^144^, ^160^, ^161^, ^162^ ^)^ The majority of these patients were found to have parathyroid adenomas or hyperplasia, whose surgical resection resulted in a decrease or normalization of serum calcium concentrations.^(^ ^144^, ^160^, ^161^, ^162^ ^)^

FHH has generally been considered a clinical diagnosis, but because of significant phenotypic overlap with typical sporadic PHPT in often‐useful measurements like the calcium:creatinine clearance ratio, germline DNA testing can play an important role in establishing or confirming the diagnosis. Making the diagnosis of FHH, with or without DNA testing, can be of major importance in clinical management, especially in highlighting the need to avoid parathyroid surgery. More specific scenarios in which DNA diagnosis can prove helpful include: individuals with FIHP; sporadic presentation of an FHH phenotype or other situations where no family members are available for evaluation; and in NSHPT where confirmation may benefit family members at risk for FHH. However, not all patients with clinically diagnosed FHH/NSHPT or FIHP will harbor a detectable germline mutation in a known causative gene. A negative test in the proband should not be used to exclude a genetic disorder. If the clinical phenotype is highly suggestive of FHH or monogenic PHPT, then further testing using a different DNA sequencing platform may be warranted.^(^ ^163^ ^)^ In addition, some patients may harbor mutations in as yet unidentified genes. Continued surveillance of mutation‐negative cases is therefore recommended. In addition, genetic testing may identify variants of unknown significance (VUS). These ambiguous findings pose a considerable diagnostic challenge. More detailed clinical phenotyping and additional testing of family members may help to clarify variant status.^(^ ^163^ ^)^

Assessment for CaSR autoantibodies should also be considered in patients with acquired hypercalcemia and hypocalciuria, particularly if there is a history or family history of autoimmune diseases.^(^ ^164^ ^)^

### Role of the CaSR polymorphisms in common forms of PHPT

In line with the identification of *CASR* mutations causing PHPT disorders with a clear Mendelian mode of inheritance, as described in previous section, common *CASR* single nucleotide polymorphisms (SNPs) may also influence the phenotype of PHPT, as they have been associated by genomewide association studies (GWASs) with circulating calcium and PTH concentrations in general outbred populations.^(^ ^165^, ^166^ ^)^ No GWASs have been performed on PHPT to date, but findings from previous association studies of limited sample size^(^ ^167^, ^168^, ^169^ ^)^ are in line with the contention that *CASR* constitutes a strong candidate gene influencing the pathogenesis of more common forms of PHPT arising in the general population. Somatic *CASR* mutations have not been detected in parathyroid tumors from PHPT patients,^(^ ^170^, ^171^, ^172^, ^173^ ^)^ and are therefore unlikely to influence their pathogenesis.

### Alteration of parathyroid CaSR expression in PHPT

**Fig. 1:**
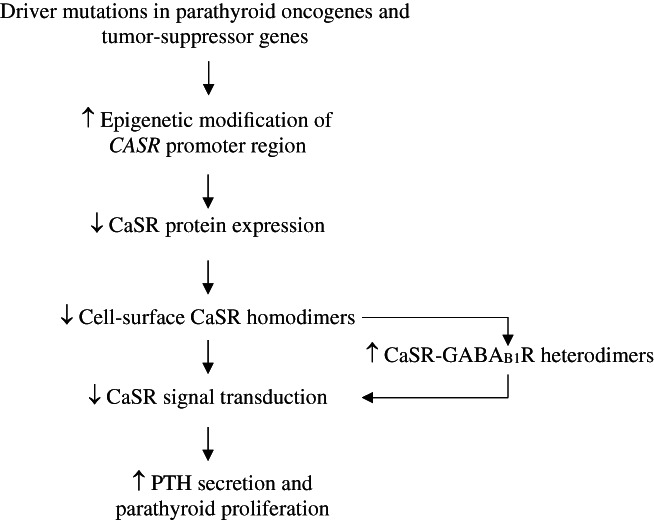
Potential role of altered parathyroid CaSR expression in the pathogenesis of PHPT. Epigenetic modification of the CASR promoter region in parathyroid adenomas may be mediated by mutations affecting oncogenes and tumor‐suppressor genes, which in turn cause reduced CaSR protein expression.^(^ ^71^ ^)^ Reduced parathyroid CaSR expression may decrease the number of functioning CaSR homodimers at the cell‐surface, which will impair CaSR signal transduction and lead to increased PTH secretion. Reduced numbers of CaSR homodimers may also cause the CaSR to form heterodimers with the parathyroid‐expressed GABA_B1_ receptor (GABA_B1_R). Such CaSR‐GABA_B1_R heterodimers are postulated to inhibit signaling from CaSR homodimers,^(^ ^175^ ^)^ thereby further increasing PTH secretion and parathyroid gland proliferation.

Alterations in parathyroid CaSR expression may contribute to PHPT pathogenesis (Fig. 1). Thus, a study of adenomatous and hyperplastic parathyroid glands from PHPT patients showed decreased CaSR expression, which was associated with an altered set‐point for Ca^++^‐mediated PTH release.^(^ ^70^ ^)^ Ultimately, such CaSR and set‐point changes may be secondary consequences of a tumor's primary driving proliferative defects, as was shown in an informative animal model of parathyroid neoplasia.^(^ ^174^ ^)^ This reduced parathyroid CaSR expression may potentially also be mediated by epigenetic modifications of the *CASR* promoter region, and increases in methylation and histone modifications (H3K9me3 and H3K27me3) of the *CASR* promoter have been reported in sporadic parathyroid adenomas with the degree of H3K9me3 modification and methylation correlating with *CASR* messenger RNA (mRNA) levels and plasma PTH concentrations, respectively.^(^ ^71^ ^)^ The cause of these epigenetic changes is unclear, but is likely mediated by driver mutations in parathyroid oncogenes and tumor‐suppressor genes (Fig. 1). Reduced CaSR expression in hyperplastic parathyroid glands from PHPT patients is also associated with increased formation of heteromeric receptor complexes comprising the CaSR and gamma amino butyric acid type B1 receptor (GABA_B1_R).^(^ ^175^ ^)^ Such heteromeric CaSR‐GABA_B1_R complexes may contribute to PTH hypersecretion in PHPT (Fig. 1).^(^ ^175^ ^)^

## Future Research and Recommendations

Recent advances described in this report have clarified certain aspects of the epidemiology, pathophysiology, and genetics of PHPT. However, there remain many unanswered questions that are recommended for future research, as detailed below.

### Epidemiology of PHPT

1. The diagnosis of asymptomatic PHPT is largely determined by the measurement of serum calcium and PTH with large variations between regions and healthcare systems. The incidence of PHPT is threefold to fivefold higher in postmenopausal women for unclear reasons, but identification of such factors may provide insight into its etiology.
2. The consequences of mild PHPT are still unclear due to disparate population‐based clinical findings including uncertainty regarding long‐term consequences of untreated disease on morbidity and mortality, which merits further investigation. This will especially be important given the increasing recognition of mild forms of PHPT in countries outside of North America and Europe.
3. There is a need for a prospective study concomitantly carried out in different parts of the world to better define prevalence and incidence of the hypercalcemic and normocalcemic forms of PHPT.

### Pathophysiological and clinical aspects of PHPT

1. The pathophysiologic basis for normocalcemic and hypercalcemic forms of PHPT requires further investigations to identify whether they are different points on a continuum of disease and/or different stages of disease evolution.
2. The breadth of pathophysiological alterations resulting in PHPT are not fully defined. Although parathyroid cell proliferation is a key cause of increased circulating PTH concentrations, the contribution of other mechanisms such as posttranscriptional processing of PTH mRNA and alterations in the parathyroid set‐point for PTH release, remain to be fully elucidated.
3. The apparent increase in the incidence of multiglandular hyperplasia in PHPT needs to be confirmed, and if proven, then possible etiological environmental and/or genetic factors need to be identified.
4. The effects of diet on progression of PHPT need to be better defined, with the aims of elucidating the effects of: dietary protein and fiber intake on the manifestations of PHPT; and dietary calcium intake on the variability in the sensitivity to PHPT.
5. The manifestations and clinical course, and yearly incidence of fractures due to PHPT in different ethnic communities need to be determined.
6. The adverse cardiovascular changes (if any) in NHPT need to be established.
7. The abnormalities in muscle function (if any) in PHPT need to be determined.
8. The adverse neuropsychiatric changes (if any) in PHPT need to be determined.

### Role of the CaSR in sporadic forms of PHPT and its physiological role in the parathyroid and kidney

1. The apparent association of some *CASR* mutations with PHPT rather than FHH, needs to be confirmed, and if proven the role of biased signaling involving proliferative pathways such as the MAPK cascade investigated.
2. The roles (if any) of *GNA11* or *AP2S1* variants in the pathogenesis of PHPT needs to be determined.
3. The CaSR signaling pathways mediating PTH secretion and parathyroid gland proliferation remain to be elucidated.
4. The contributions of the kidney CaSR to systemic calcium homeostasis requires further research, and in particular its interaction with PTH in regulating plasma calcium concentrations and urinary calcium excretion.

### Genetics of PHPT

1. Determining, by use of Grading of Recommendations, Assessment, Development, and Evaluations (GRADE) methodology,^(^ ^176^ ^)^ the effects of genetic testing in altering management/treatment/outcomes in different groups of PHPT patients, eg, young (<35 years of age) and/or those with: family history of PHPT (or endocrine neoplasia); multigland (parathyroid) disease; parathyroid carcinoma; and other endocrine tumors.
2. Application of GRADE^(^ ^176^ ^)^ methodology to evaluate utility of genetic testing modalities (eg, single gene testing, hyperparathyroid gene panel [*MEN1*, *RET*, *CaSR*, *CDC73*, *CDKNK1B*, *AP2S1*, *GNA11*] testing, exome sequencing, or whole genome sequencing) for identifying mutations in patients with PHPT.
3. Identification and/or improved clinical and molecular understanding of additional causative or contributory genes for familial PHPT, syndromic or nonsyndromic, and identification of less penetrant modifier genes for all forms of FIHP.

## Author Contributions

**Salvatore Minisola:** Conceptualization; writing – review and editing. **Andrew Arnold:** Data curation; formal analysis; investigation; methodology; writing – original draft; writing – review and editing. **Zhanna Belaya:** Data curation; formal analysis; investigation; methodology; writing – original draft; writing – review and editing. **Maria Luisa Brandi:** Funding acquisition; writing – review and editing. **Bart L. Clarke:** Funding acquisition; writing – review and editing. **Fadil M Hannan:** Data curation; formal analysis; investigation; methodology; writing – original draft; writing – review and editing. **Lorenz Hofbauer:** Data curation; formal analysis; investigation; methodology; writing – original draft; writing – review and editing. **Karl L. Insogna:** Data curation; formal analysis; investigation; methodology; writing – original draft; writing – review and editing. **André Lacroix:** Writing – review and editing. **Uri Aharon Liberman:** Writing – review and editing. **Andrea Palermo:** Data curation; formal analysis; investigation; methodology; writing – original draft; writing – review and editing. **Jessica Pepe:** Writing – review and editing. **René Rizzoli:** Data curation; formal analysis; investigation; methodology; writing – original draft; writing – review and editing. **Robert Alan Wermers:** Data curation; formal analysis; investigation; methodology; writing – original draft; writing – review and editing. **Rajesh V. Thakker:** Conceptualization; writing ‐ review and editing.

## Conflicts of Interest

We acknowledge unrestricted financial support from: Amolyt, Ascendis, Calcilytix and Takeda. They had no input into the planning or design of the project, the conduct of the reviews, evaluation of the data, writing or review of the manuscript, its content, conclusions, or recommendations contained herein. SM served: as speaker for Abiogen, Amgen, Bruno Farmaceutici, Diasorin, Eli Lilly, Shire, Sandoz, Takeda; and on advisory board of Abiogen, Kyowa Kirin, Pfizer, UCB. LCH has received honoraria for clinical trials to his institutions from Amgen.

## Ethical Statement

These papers are retrospective reviews and did not require ethics committee approval.

### Peer Review

The peer review history for this article is available at https://publons.com/publon/10.1002/jbmr.4665.

## Data Availability

The data that support the findings in this study are openly available in PubMed, MEDLINE, EMBASE, and the Cochrane databases.
